# The Participation of Calponin in the Cross Talk between 20-Hydroxyecdysone and Juvenile Hormone Signaling Pathways by Phosphorylation Variation

**DOI:** 10.1371/journal.pone.0019776

**Published:** 2011-05-19

**Authors:** Peng-Cheng Liu, Jin-Xing Wang, Qi-Sheng Song, Xiao-Fan Zhao

**Affiliations:** 1 The Key Laboratory of Plant Cell Engineering and Germplasm Innovation, Ministry of Education, School of Life Sciences, Shandong University, Jinan, China; 2 Division of Plant Sciences, University of Missouri, Columbia, Missouri, United States of America; New Mexico State University, United States of America

## Abstract

20-hydroxyecdysone (20E) and juvenile hormone (JH) signaling pathways interact to mediate insect development, but the mechanism of this interaction is poorly understood. Here, a calponin homologue domain (Chd) containing protein (HaCal) is reported to play a key role in the cross talk between 20E and JH signaling by varying its phosphorylation. Chd is known as an actin binding domain present in many proteins including some signaling proteins. Using an epidermal cell line (HaEpi), *HaCal* was found to be up-regulated by either 20E or the JH analog methoprene (JHA). 20E induced rapid phosphorylation of HaCal whereas no phosphorylation occurred with JHA. HaCal could be quickly translocated into the nuclei through 20E or JH signaling but interacted with USP1 only under the mediation of JHA. Knockdown of *HaCal* by RNAi blocked the 20E inducibility of *USP1*, *PKC* and *HR3*, and also blocked the JHA inducibility of *USP1*, *PKC* and *JHi*. After gene silencing of *HaCal* by ingestion of *dsHaCal* expressed by *Escherichia coli*, the larval development was arrested and the gene expression of *USP1*, *PKC*, *HR3* and *JHi* were blocked. These composite data suggest that HaCal plays roles in hormonal signaling by quickly transferring into nucleus to function as a phosphorylated form in the 20E pathway and as a non-phosphorylated form interacting with USP1 in the JH pathway to facilitate 20E or JH signaling cascade, in short, by switching its phosphorylation status to regulate insect development.

## Introduction

Protein phosphorylation and dephosphorylation are recognized as a key mechanism in various signaling transductions [1]. However, the phosphorylation and the involved molecules in the signaling transduction induced by hormones are not fully understood. Insect growth and development are regulated by two major hormones: the steroid 20-hydroxyecdysone (20E) and the sesquiterpenoid juvenile hormone (JH). 20E initiates and orchestrates molting and metamorphosis [2], [3], [4], [5]. JH mediates the molting direction to the next larval instar by modifying or switching the expression of genes that are involved in the 20E signaling transduction, thus preventing premature metamorphosis at larval stages [4], [5]. The balance between these two hormones determines the scenario of insect development [6]. This offers us a good model to investigate the phosphorylation and mechanisms of hormonal signaling and cross talk.

Different mechanisms of 20E and JH cross talk have been hypothesized. The ecdysone receptor (EcR) and USP form a heterodimeric protein complex, which serves as the 20E receptors to facilitate the initiation of the downstream transcriptional cascade by 20E [7]. Li et al. (2007) presented a model for insect hormone signaling pathways based on the in vitro yeast hybridization results, in which a Chd-containing protein Chd64 in *Drosophila melanogaster* is identified as a chaperone protein that can bind to other proteins including USP in *Drosophila*. Another *Drosophila* Chd-containing protein DroVav was suggested to play a pivotal role as a signal transducer protein by associating it with tyrosine-phosphorylated epidermal growth factor receptor during fruit fly development [8]. The Chd is known as actin binding domain, present in cytoskeletal proteins and some signaling proteins [9]. However, the function of the Chd is not well understood.

Our previous findings showed that a Chd-containing protein HaCal was up-regulated during metamorphosis and phosphorylated by PKC under 20E stimulation when the differentially expressed phosphoprotein profiles were examined during the *Helicoverpa armigera* metamorphosis [10]. In the present study, we found that *HaCal* was expressed throughout the development and was up-regulated and quickly translocated into the nuclei by both 20E and methoprene. HaCal was phosphorylated through PKC by 20E induction and not by methoprene. The non-phosphorylated form of HaCal interacted with USP1 and played a key role as an intermediary or feedback regulator in the cross talk between the 20E and JH signaling transduction pathways. The transcription of *HaCal* was in turn up-regulated by 20E and JH, respectively, through the network of *EcR-B1*, methoprene-tolerant (*Met1*), *PKC,* or Broad-Complex (*Br-Z2*). Gene silencing of *HaCal* in *H. armigera* larvae led to the retarded larval growth and abnormal molting, resulting in the defective larval development.

## Results

### *HaCal* was highly expressed at the feeding, metamorphic, and pupal stages

**Figure 1:**
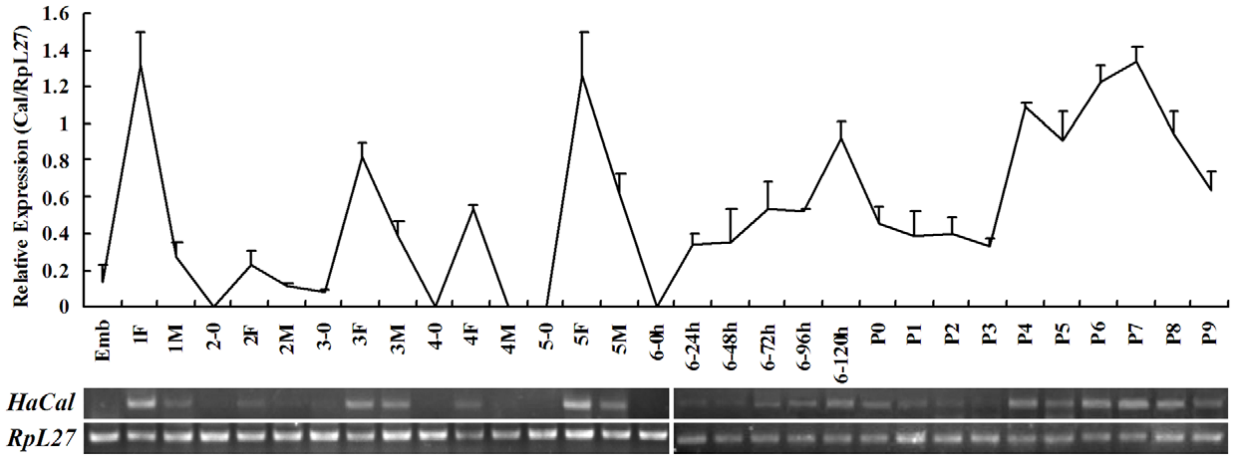
Semi-quantitative RT-PCR analysis of the developmental changes in *HaCal* mRNA levels. *RpL27* RNA was used as a quantitative control. About 3–20 insects were pooled for each sample at different stages. The values are mean ± S.D. The experiments were repeated at least three times and each was performed in triplicate. Emb: embryos; 1F, 2F, 3F, 4F, 5F: the first to fifth instar feeding larvae; 1 M, 2 M, 3 M, 4 M, 5 M: the first to fifth instar molting larvae; 6–24 h, 6–48 h: sixth instar feeding larvae; 6–72 h, 6–96 h, 6–120 h: sixth instar metamorphic-committed larvae; P0–P9: pupae on day 0 to day 9.

To understand the function of *HaCal*, its expression patterns during development from larvae to preadult were examined initially. The level of *HaCal* mRNA exhibited several distinguishable peaks throughout the development of *H. armigera*. During the feeding stages of the first, second, third, fourth and fifth instar larvae, *HaCal* showed a relatively higher level of transcript than that in each molting stage. During the final instar feeding stage, *HaCal* was lower but it slightly increased from 6–72 h and peaked at 6–120 h just before pupation, in which the larvae were metamorphically committed. *HaCal* increased again during adult development from mid-pupal stage to preadult (Fig. 1). This expression pattern of *HaCal* mRNA suggests that it plays a role during larval growth and metamorphosis.

### *HaCal* was up-regulated by either 20E or methoprene (JHA)

**Figure 2:**
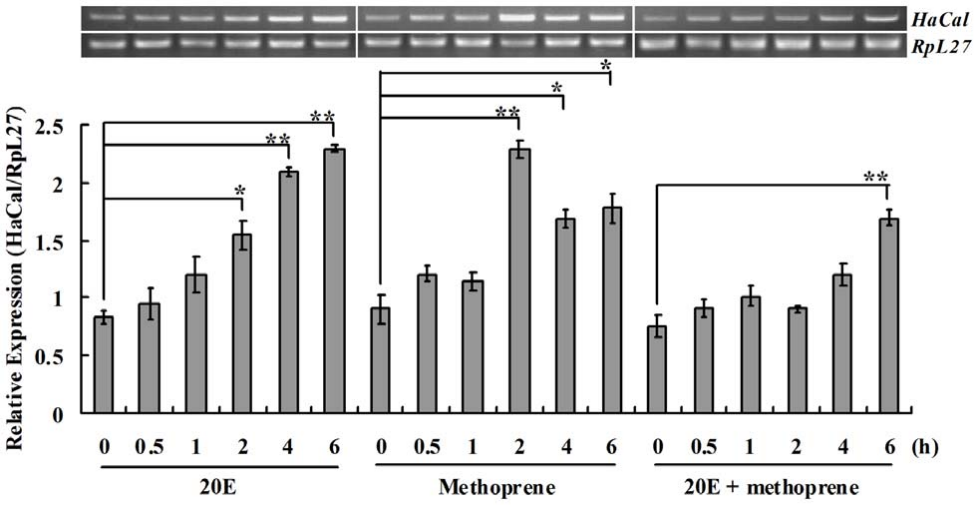
Semi-quantitative RT-PCR analysis of the hormone regulation of *HaCal* in HaEpi cells. 20E or methoprene was added to the cells to obtain a final concentration of 1 µM. The cells were then cultured for 0.5, 1, 2, 4, and 6 h, respectively, and RNA was extracted for RT-PCR by agarose gel electrophoresis. The values are mean ± S.D. (*n* = 3). * denoted significant difference (p<0.05), ** denoted significant difference (p<0.01). See also Fig. S1.

The developmental expression profile of *HaCal* seemed related to the titer variation of hormones in *H. armigera*, which promoted us to investigate further its hormonal regulation. An epidermal cell line of *H. armigera* (HaEpi) [11] was treated with 20E or the JH analog methoprene. Results showed that 20E caused a steady increase in *HaCal* beginning at 4 h, whereas methoprene caused a peak of expression at 2 h. The application of 20E and methoprene delayed the hormonal induction of *HaCal* mRNA until 6 h (Fig. 2). Thus, *HaCal* was induced by both 20E and JH analog methoprene in HaEpi cells, respectively. In addition, 20E response gene hormone receptor 3 (*HHR3*) was up-regulated 1 h upon 20E treatment. The mRNA level of JH inducible gene (*JHi*), which was proved to be a secondary JH response gene in *Drosophila* [12], was elevated 4 h post methoprene treatment. This indicated that the cell line responds to the hormones well (Fig. S1).

### HaCal was phosphorylated by 20E signaling and not by JH signaling

**Figure 3:**
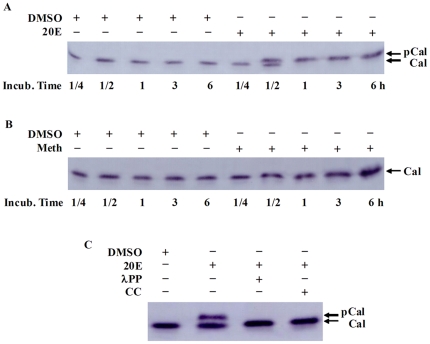
Western blot analysis of hormone induction and phosphorylation of HaCal in HaEpi cells. A, Cultured cells were added with 20E to a final concentration of 1 µM, incubated for 15 and 30 min as well as 1, 3, and 6 h, respectively. Control cells received equal volumes of DMSO. B, Cultured cells were added with methoprene (Meth) to a final concentration of 1 µM. C, The cells were treated with 20E for 30 min, and then the protein was extracted and the partial sample was incubated with λ protein phosphatase (λPP). In addition, protein kinase C specific inhibitor CC (5 µM final concentration) was added to cells shortly before 20E application and incubated for an additional 30 min.

In order to reveal the difference between the 20E and methoprene induction of HaCal, the protein expression levels of HaCal were examined in HaEpi cells after treatments by 20E or methoprene. Results showed that both 20E and methoprene up-regulated the expression of HaCal. However, a significant difference was that 20E resulted in the increase of the molecular weight of HaCal post treatment for 30 min (Fig. 3A) while methoprene kept the molecular weight constant (Fig. 3B).

In order to confirm whether the difference between the two forms of HaCal caused by 20E treatment resulted from post-translational modification such as phosphorylation, the same protein sample from the 20E-treated cells was incubated with Lambda protein phosphatase (λPP). Results showed that after λPP treatment, the upper band diminished and the lower band increased correspondingly. This demonstrated that the migration difference between the larger molecular weight band and the smaller molecular weight band of HaCal is due to the phosphorylation modification. Thus, the upper band was the phosphorylated form of HaCal and the lower band was the dephosphorylated form. When the PKC specific inhibitor CC was added to the cells, 20E could no longer induce the upper band but only increase the lower band signal accordingly (Fig. 3C). These results indicated that protein kinase C was responsible for the 20E-induced HaCal phosphorylation.

### 20E or JHA directed the nuclear translocation of HaCal in HaEpi cells

**Figure 4:**
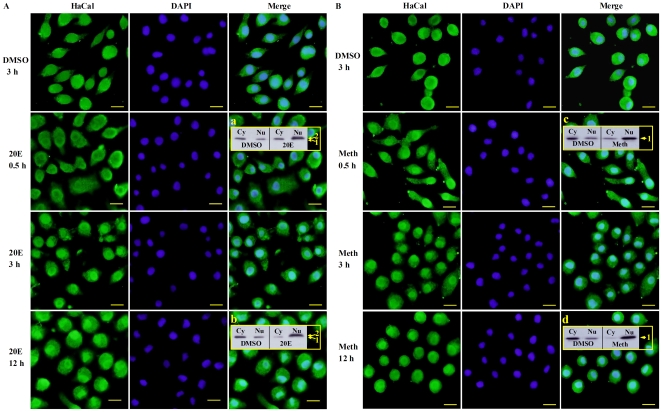
Immunocytochemistry to analyze the effects of 20E (A) and methoprene (B) on HaCal subcellular localization. Cultured cells were incubated with 20E or methoprene, and then immunostained with anti-HaCal antibody (green color). Nuclei of the cells were stained with DAPI (blue color). HaCal signal was visualized using an Olympus BX51 fluorescence microscope. At least 3 biological replicates were performed and the images are typical ones. a, b, c, and d confirming the subcellular localization of HaCal in the cytoplasm and nucleus by Western blot. Arrow 1 indicates the non-phosphorylated HaCal, and arrow 2 indicates the phosphorylated HaCal. Cy: cytosolic fraction. Nu: nuclear fraction. The yellow bar denotes 20 µm at 40 × magnifications.

HaEpi cells were treated with 20E or methoprene and analyzed by immunocytochemistry to investigate whether the phosphorylated HaCal and the non-phosphorylated HaCal mediated by 20E and methoprene, respectively, had different subcellular localizations, and to probe further the action of HaCal in vivo. Results showed that a relatively even level of HaCal was maintained in both cytoplasm and nucleus in the absence of either 20E or methoprene (Fig. 4). However, the dynamic equilibrium of HaCal between different intracellular locations changed after the addition of 20E to the cells. HaCal started to migrate into the nuclei half an hour post 20E treatment and its nuclear accumulation increased with time. Firstly, it accumulated around the inner side of the karyotheca in the nuclear zone. Then, HaCal increased in the nucleus 3 h later, and HaCal reached a considerably high level in the nuclei till 12 h (Fig. 4A).

Interestingly, methoprene treatment on the HaEpi cell line also resulted in a similar nuclear translocation of HaCal, except that HaCal greatly accumulated the nuclei at 0.5 h, which was a little faster than the 20E treatment (Fig. 4B). These evidences indicated that both 20E and methoprene can regulate the nuclear translocation of HaCal in HaEpi cells. However, the subcellular localization of HaCal was not directly related to the variation of its phosphorylation status.

### Methoprene-mediated non-phosphorylated HaCal binds to HaUSP1

**Figure 5:**
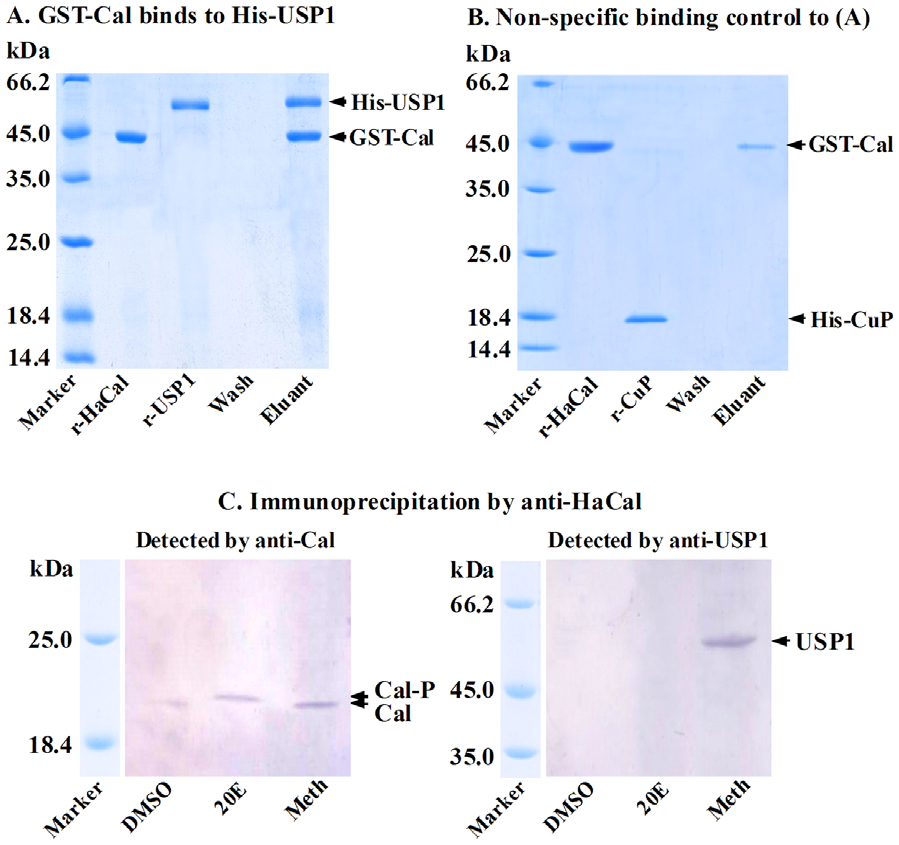
Protein interaction between HaCal and USP1. A, In vitro binding. Recombinant GST-fusion HaCal (GST-Cal) was loaded to the glutathione Sepharose 4B Resin as a bait protein. The recombinant USP1 (His-USP1) was then incubated as a prey. After being washed with 15 mL PBS, the bound proteins were eluted with an elution buffer and separated by 12.5% SDS-PAGE and stained with Commassie blue. B, Recombinant cuticle protein (His-CuP) was used as a negative control for (A). C, In vivo binding of HaCal and USP1. HaEpi cells were incubated with 20E or methoprene. Control cells received equal dilution of DMSO. Total proteins from the treated cells were extracted and precipitated by polyclonal antibody against HaCal through CNBr-activated Sepharose 4B beads. The bound proteins were eluted from the Sepharose beads after extensive washing and subjected to Western blot analysis using antibodies against HaCal and USP1, respectively.

To understand further the roles of HaCal in the nuclei following the induction of 20E and methoprene, the interaction between HaCal and USP1 was investigated. Results in vitro showed that when the recombinant His-USP1 flowed through the GST-HaCal bond resins, His-USP1 was co-eluted with GST-HaCal in the eluant (Fig. 5A). In contrast, by the same method, the recombinant cuticle protein His-CuP was not eluted together with GST-Cal in the control (Fig. 5B). This result demonstrated that the non-phosphorylated HaCal can bind to the non-phosphorylated HaUSP1 expressed in *E. coli* in vitro.

Further immunoprecipitation (IPP) experiments confirmed that methoprene mediated the interaction between HaCal and USP1 in vivo. When the cells were treated with 20E, USP1 cannot be co-immunoprecipitated by anti-HaCal antibodies. However, in contrast, when the cells were treated with methoprene, USP1 can be detected in the precipitate (Fig. 5C). These findings showed that only nonphosphorylated HaCal interacted with USP1 when induced by methoprene, but phosphorylated HaCal cannot interact with USP1 when induced by 20E. Thus, HaCal participated in the 20E and JH signaling pathways with its phosphorylation status being alternatively changed.

### HaCal was necessary for either 20E or JH signaling transduction in HaEpi cells

**Figure 6:**
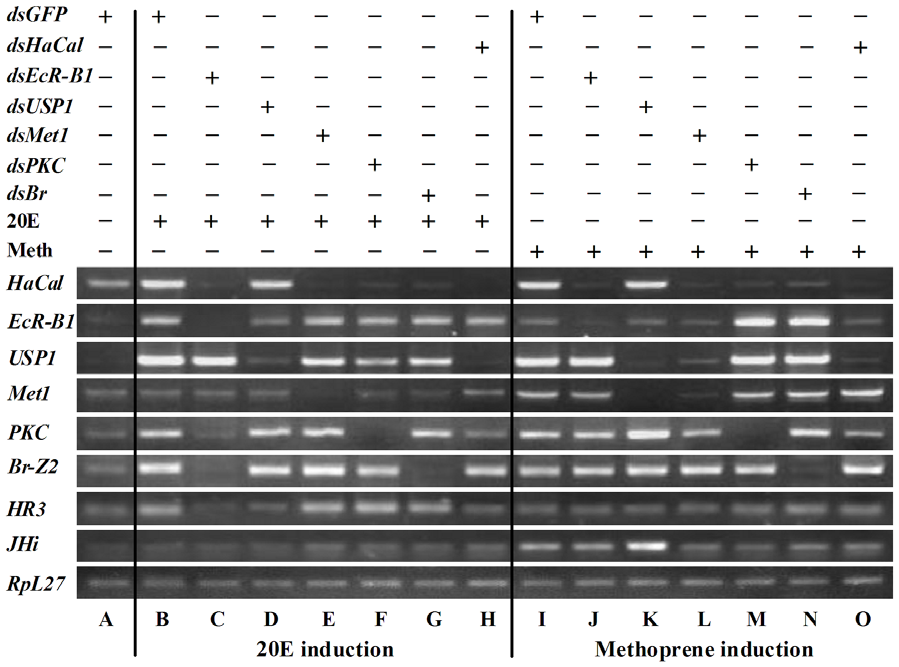
RNAi analysis of the role of *HaCal* in hormone signaling by RT-PCR in HaEpi cells. Cells were transfected with dsRNAs of *EcR-B1*, *USP1*, *Met1*, *PKC*, *Br-Z2*, and *Cal*, respectively, and then treated with 20E or methoprene (Meth) for 12 h at a final concentration of 1 µM, respectively. Total RNA was isolated and finally subjected to RT-PCR analysis by agarose gel electrophoresis. An equivalent volume of DMSO was used as solvent control for 20E and methoprene, dsGFP as a non-specific dsRNA control, and *RpL27* as a quantitative control. See also Fig. S2.

Based on the acquired results, HaCal was up-regulated and translocated into the nuclei by both 20E and methoprene, HaCal was phosphorylated unable to bind USP1 in the presence of 20E while non-phosphorylated HaCal interacted with USP1 after methoprene induction. We assumed that HaCal was involved in the regulation of the cross talk between the 20E and JH signaling pathways. To prove this hypothesis, we knocked down *HaCal* in the HaEpi cells by RNAi and checked the gene expression of *EcR-B1*, *USP1*, *Met1*, *PKC*, *Br-Z2*, *HR3*, *JHi* and *HaCal* (Fig. 6). EcR-B1 and USP1 formed heterodimer as 20E receptor. Met1 acted as JH receptor candidate. PKC-mediated phosphorylation was critical for hormonal signaling. Br was likely to mediate the hormonal interaction. HR3 and JHi served as indicators separately responded to 20E or JH. All these genes of vital importance were adopted for checking the effect of *HaCal* silencing on hormonal pathways. There may be hundreds of other genes not yet studied as far as 20E and JH dependence is concerned.

Results showed that in the absence of 20E or methoprene, the transcript levels of the genes involved in the 20E or JH pathways, including 20E receptor *EcR-B1* and its heterodimmer *USP1*, JH receptor candidate *Met1,* protein kinase C *PKC*, transcription factor *Br-Z2* and *HR3*, *HaCal*, and *JHi*, were relatively low (Fig. 6A, lane A; Fig. S2A, B). After the treatment of cells with 20E, mRNAs of *EcR-B1*, *USP1*, *PKC*, *Br-Z2*, *HaCal*, and *HR3* evidently increased (lane B compared to A). Likewise, treatment of methoprene for 12 h also elevated the transcript levels of *Met1*, *USP1*, *PKC*, *Br-Z2*, *HaCal*, and *JHi* in HaEpi cells (lane I compared to A). These indicated that *EcR-B1* and *HR3* were induced only by 20E while *Met1* and *JHi* were induced only by methoprene. *USP1*, *PKC*, *Br-Z2*, and *HaCal* were induced by both 20E and methoprene.

However, knockdown of *HaCal* by RNAi caused the suppression of transcript levels of *USP1*, *PKC*, and *HR3* but not of *EcR-B1* and *Br-Z2* induced by the 20E signaling (lane H compared to B). Simultaneously, RNAi of *HaCal* suppressed the transcript levels of *USP1, PKC*, and *JHi* but not of *Met1* and *Br-Z2* induced by methoprene in the JH signaling (lane O compared to I). In contrast, knockdown of *EcR-B1*, *Met1*, *PKC*, or *Br-Z2* suppressed the transcript level of *HaCal* induced by 20E (lane C, E, F, and G compared to B) or by methoprene (lane J, L, M, and N compared to I). These data suggest that *HaCal* sits upstream of *USP1*, *HR3*, and *JHi*, at the transcript levels, but downstream of *EcR-B1*, *Met1*, and *Br-Z2. PKC* and *HaCal* might regulate with each other on the transcript levels in the 20E and JH pathways.

### EcR-B1, USP1, Met1, PKC, and Br-Z2 formed a network involved in both 20E and JH signaling pathways

The gene network involved in the 20E or JH signaling pathway was examined further (Fig. 6; Fig. S2). Results showed that knockdown of *EcR-B1* blocked the transcript accumulation of *PKC*, *Br-Z2*, *HaCal*, and *HR3* induced by 20E (Fig. 6, lane C compared to B). Knockdown of *EcR-B1* also blocked the induction of *HaCal* by methoprene. However, the transcript levels of *USP1*, *PKC*, and *Br-Z2* induced by methoprene were not suppressed (lane J compared to I). This indicated that *EcR-B1* was necessary in the 20E and JH pathways for regulating *HaCal* expression on mRNA levels, but was not necessary for regulating *USP1, PKC*, and *Br-Z2* by methoprene.

Knockdown of *USP1* partially suppressed the induction of *EcR-B1* and *HR3* but did not suppress the mRNA level of *HaCal* induced by 20E (lane D compared to B). Knockdown of *USP1* also did not suppress the level of *HaCal* mRNA but suppressed the level of *Met1* and increased the levels of *PKC* and *JHi* in the JH signaling (lane K compared to I). These results indicated that *USP1* was not involved in regulating *HaCal* mRNA accumulation, but was involved in the induction of *EcR-B1* and *HR3* by 20E, and induction of *Met1* by methoprene.

Knockdown of *Met1* did not block *EcR-B1*, *PKC*, *Br-Z2*, and *HR3* but partially blocked the transcript levels of *USP1* and *HaCal* induced by 20E (lane E compared to B). Knockdown of *Met1* suppressed the transcript levels of *USP1*, *PKC*, *HaCal*, and *JHi* induced by methoprene (lane L compared to I). These results suggested that *Met1* was involved in the 20E and JH pathways by regulating the transcription of *USP1* and *HaCal*.

Knockdown of *PKC* suppressed the transcript levels of *USP1*, *Br-Z2*, and *HaCal* induced by 20E (lane F compared to B). Knockdown of *PKC* also suppressed the mRNA level of *HaCal* but increased the mRNA level of *EcR-B1* induced by methoprene (lane M compared to I). These results suggested that *PKC* was necessary in the 20E and JH pathways in the induction of *USP1*, *Br-Z2*, and *HaCal*, but was also a suppressor of *EcR-B1* in the JH pathway.

Knockdown of *Br-Z2* blocked the transcript levels of *USP1* and *HaCal* induced by 20E (lane G compared to B). However, knockdown of *Br-Z2* did not block the level of *USP1* transcript but increased the level of the *EcR-B1* transcript induced by methoprene (lane N compared to I). These results suggested that *Br-Z2* was necessary for the 20E pathway in regulating the transcript accumulation of *USP1* and *HaCal*, and it played a role to suppress the 20E pathway in the presence of methoprene.

These composite results suggested that EcR-B1, Met1, USP1, PKC, and Br-Z2 formed an interactive network involved in both 20E and JH pathways. They played roles either in the 20E or JH pathway by regulating different gene expressions at the related developmental stages. Their functions might be regulated by other chaperone protein suchs as HaCal.

### Knockdown of *HaCal* in larvae arrested the larval development

To investigate the role of *HaCal* in the developmental process of *H. armigera*, *HaCal* was knocked down by feeding the larvae with bacteria that expressed *dsHaCal*. Epidermis, midgut, fat body and hemocytes, which responded to 20E or JH well in *H. armigera* [13], [14] and were also easily accessible for detailedly investigating gene transcription, were dissected after RNAi in larvae. The total RNA was extracted and finally subjected to RT-PCR analysis.

**Figure 7:**
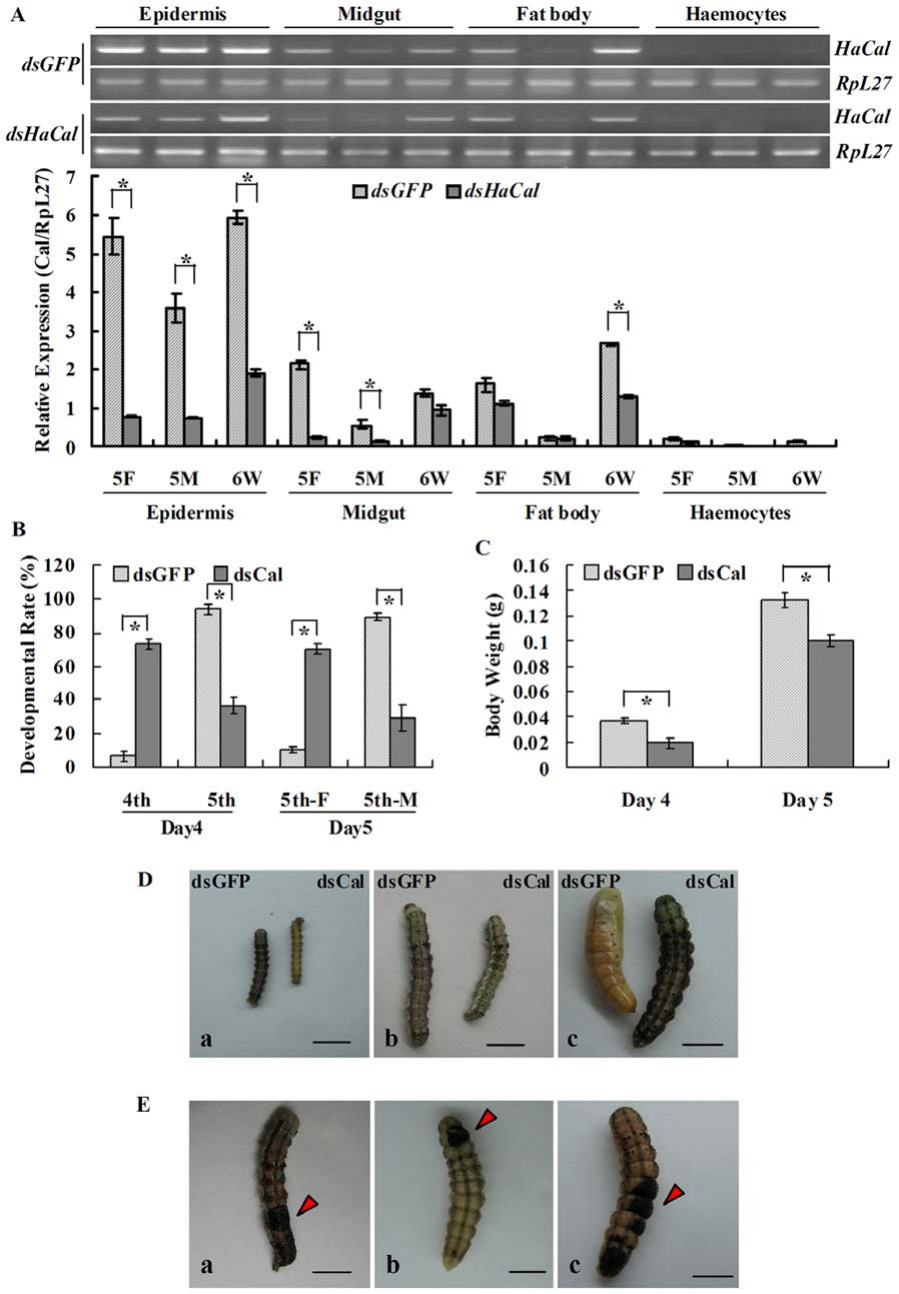
Effect of *HaCal* silencing on larval development of *H. armigera*. A, Semi-quantitative RT-PCR analysis of transcript levels of *HaCal* after the larvae ingested bacterially expressed *dsHaCal*. 5F, 5 M, and 6 W denote 5th instar feeding larvae, 5th instar molting larvae, and 6th instar wandering larvae, respectively. * denotes the significant difference (p<0.05, by student *t* test). *RpL27* was used as a reference. B, Delayed development after knockdown of *HaCal*. C, Decreased body weight after knockdown of *HaCal*. D, Postponed body size after knockdown of *HaCal*. E, Abnormalities after knockdown of *HaCal*. *EcR-B1*, *USP1*, *Met1*, *PKC*, *Br-Z2*, *Cal, HR3,* and *JHi*.

Results showed that the mRNA levels of *HaCal* in epidermis were substantially reduced after continuous feeding with *dsHaCal* as compared to the control group with *dsGFP* at the 5th instar feeding stage (5F), 5th instar molting stage (5M), and 6th instar metamorphically-committed stage (wandering stage, 6W). The mRNA levels of *HaCal* in the midgut at 5F and 5M as well as in the fat body at 6W were also brought down after feeding with the bacterially expressed *dsHaCal*. Little mRNA accumulation was detected in hemocytes at these typical developmental stages after feeding with either *dsGFP* or *dsHaCal* (Fig. 7A).

The knockdown of the *HaCal* retarded larval growth of *H. armigera*, which was accompanied by gene silencing following the ingestion of bacteria-expressed *dsHaCal*. On day 4 post-feeding, 94% of the larvae in the *dsGFP* feeding group entered the 5th instar while 73% of the larvae in the *dsHaCal* feeding group remained in the 4th instar feeding stage. On day 5, 90% of the *dsGFP*-fed larvae were molting towards the 6th instar while 70% of the *dsHaCal-*fed larvae were still in the 5th instar feeding stage (Fig. 7B). Correspondingly, the larvae fed with *dsHaCal* clearly had a lower body weight and a smaller body size than the larvae fed with *dsGFP* on day 4 and day 5 of post-feeding (Fig. 7C and D), and formed a smaller pupae. In addition, about 9% of the larvae fed with *dsHaCal* failed to complete the molt with a “half-ecdysis” phenotype surviving for 24 h and then dying. Some of the larvae formed blackened spots on the cuticle (Fig. 7E). These data suggested that the larval development of *H. armigera* was retarded, which was resulted from two developmental defects, the slowed larval growth and the delayed larval molting (Fig. 7B-E).

### Knockdown of *HaCal* in larvae suppressed the 20E or JH signaling

**Figure 8:**
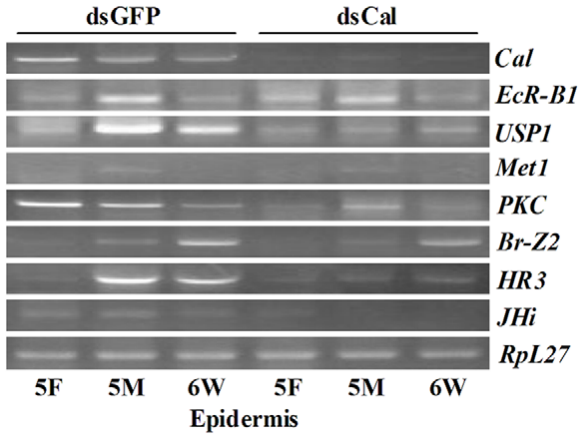
Effect of *HaCal* silencing on the 20E or JH response genes analyzed by RT-PCR. cDNAs from the epidermis of larvae, in which *HaCal* was silenced by ingestion of bacterially expressed ds*HaCal*, were used to check the RNAi effect on these key factors involved in 20E and/or JH signaling pathways. See also Fig. S3.

There was interest in exploring the underlying molecular foundation behind the slowed larval growth and delayed larval molting after knockdown of *HaCal*. This was done by detecting the transcript levels of the genes that may participate in the 20E or JH signaling transduction pathway. Results showed that in the epidermis after the *HaCal* silencing, the mRNA accumulation of *USP1* and *JHi* at all three stages as well as *PKC* in 5F and *HR3* during molt and metamorphosis were suppressed (Fig. 8, Fig. S3). These results were consistent with the results from the cell line after knockdown of *HaCal*. These confirmed in vivo that HaCal was necessary for gene transcription in the cross talk between the 20E and JH signaling transduction pathways and thus played key roles in the development of *H. armigera*.

## Discussion

In this study, calponin (HaCal) was shown to be present, albeit at fluctuating levels, throughout the development of *H. armigera.* Its suppression by HaCal RNAi caused both the retarded and reduced larval growth. Using an epidermal cell line, HaCal was found to be regulated by both 20E and the JH analog methoprene. Both caused its up-regulation and migration into the nucleus, but only 20E caused its phosphorylation via the PKC pathway. In these cells and based on its suppression by RNAi, this phosphorylated calponin was necessary for the up-regulation of USP1 and HR3 as well as part of the up-regulation of PKC by 20E. In the methoprene-treated cells, the non-phosphorylated calponin bound with USP1 was necessary for methoprene-induced USP1 up-regulation along with that of JHi. Thus, HaCal is critical in both the 20E and the JH signaling pathways and likely mediates their cross talk.

### HaCal is regulated by JH and 20E

The calponin homologue domain (Chd) is known as actin binding domain, present in cytoskeletal proteins such as transgelin, calponin, spectrin and dystrophin/utrophin, and also present in some signaling proteins such as human GTPase activating protein of Ras (Ras-GAP) and Vav [15]. Human Ras-GAP contains a Chd capable of interacting with an intricate network of cellular enzymes and structures. Ras-GAP affects the GTPase activity, nucleotide exchange rates and membrane localization of Ras superfamily members, thus participates in the regulation of cell growth, differentiation and action [16], [17]. Human Vav is a proto-oncogene product and a complex modular protein which is specifically expressed in hematopoietic cells related to signaling transduction and essential for normal function of these cells [18], [19]. However, in addition to the function of actin binding, other function of Chd in these proteins is unknown.

Here in *H. armigera*, HaCal contains only a single Chd and shares relatively a high identity with Chd64 in *Drosophila* (67%). *Drosophila* Chd64 seems prevalent throughout the larval growth but lowly expresses during the final instar in *Drosophila*. Chd64 can be up-regulated by exogenous JH III [20]. In *Helicoverpa*, our findings showed that HaCal exhibited high expression levels during the final instar at the metamorphic stage and pupal stages not only at the larval feeding stages. HaCal was regulated by JH and 20E in vivo. *H. armigera* has similar developmental stages to *M. sexta* [13], in which the JH titer is higher at the feeding stage and decreases during molting and metamorphosis while 20E dominates the latter stages. This hypothesis is confirmed by the induced transcript abundance of HaCal by exogenous applications of both 20E and the JH analog methoprene in HaEpi cells. The different expression patterns of this gene in *Drosophila* and *Helicoverpa* may reflect the differences in hormonal regulation in the two species. The sequence alignment and phylogenetic tree analysis of HaCal with Chd-containing proteins of other species was supplied in supporting information (See Fig. S4, S5 and S6).

### HaCal plays roles by varying its phosphorylation under hormone regulation

Although both 20E and the JH analog methoprene up-regulate the expression of HaCal, the post-translational modifications of HaCal mediated by 20E and methoprene are shown to be different. 20E signaling leads to HaCal phosphorylation while methoprene does not. During larval feeding when the JH titer is high, HaCal plays a role in a non-phosphorylated form. However, at the metamorphic and pupal stages when the 20E titer is high, HaCal plays a role in a phosphorylated form.

Protein kinase C (PKC) has been reported to be involved in various membrane signaling pathways [21], [22] and plays a key role in regulating insect growth and development [23]. PKC is shown to be involved in the phosphorylation of USP [24] through 20E signaling. Moreover, PKC-mediated phosphorylation suppresses JH action by preventing nucleus protein binding to JH response elements [25]. Our work has revealed that HaCal is phosphorylatd by PKC, which supports the finding that calponin co-immunoprecipitated with protein kinase C-epsilon [26]. Our evidence also indicates that PKC is necessary for the transcript accumulation of *HaCal* induced by 20E or methoprene. Moreover, knockdown of *PKC* increases the methoprene inducibility of *EcR-B1*, suggesting that PKC acted as a suppressor of EcR-B1 in the JH pathway.

### 20E and JH interact through a protein network

Several theories have been put forward to explain the scenario of cross talk between 20E and JH signaling [27]. The network among the factors and their roles in insect development became more mysterious and attractive along with deep investigation. Quite a few studies have shown that JH and 20E may cross talk via a set of transcriptional regulators, such as EcR, HR3, Met, JHi, USP, Br, and Chd64. EcR and USP form a heterodimeric protein complex, which serves as the 20E receptors to facilitate the initiation of the downstream transcriptional cascade by 20E [7]. USP phosphorylation mediated by PKC is required for 20E-induced gene expression [24], [28]. HR3 is a nuclear hormone receptor and an early-late indicator of 20E signaling pathway that is up-regulated by 20E [14], [29]. USP and Met are the candidate receptors of JH [27], [30], [31], [32], [33] even though USP binds JH with low affinity and the loss of Met does not cause larval death. Met acts upstream of Br in JH signaling to preclude metamorphosis in *Tribolium castaneum* [34], [35]. Our data showed that USP1 or Met was necessary for different gene transcription by JH induction. Br is involved in the cross talk between the 20E and JH signaling pathways [30], [36]. In the epidermis of *Manduca,* Br mRNA is directly induced by 20E [37]. JH application at the onset of pupal to adult transition in *Tribolium* leads to Br re-expression [35], [37]. JHi is a JH inducible gene [38], which can be used as an indicator for the JH signaling pathway.

**Figure 9:**
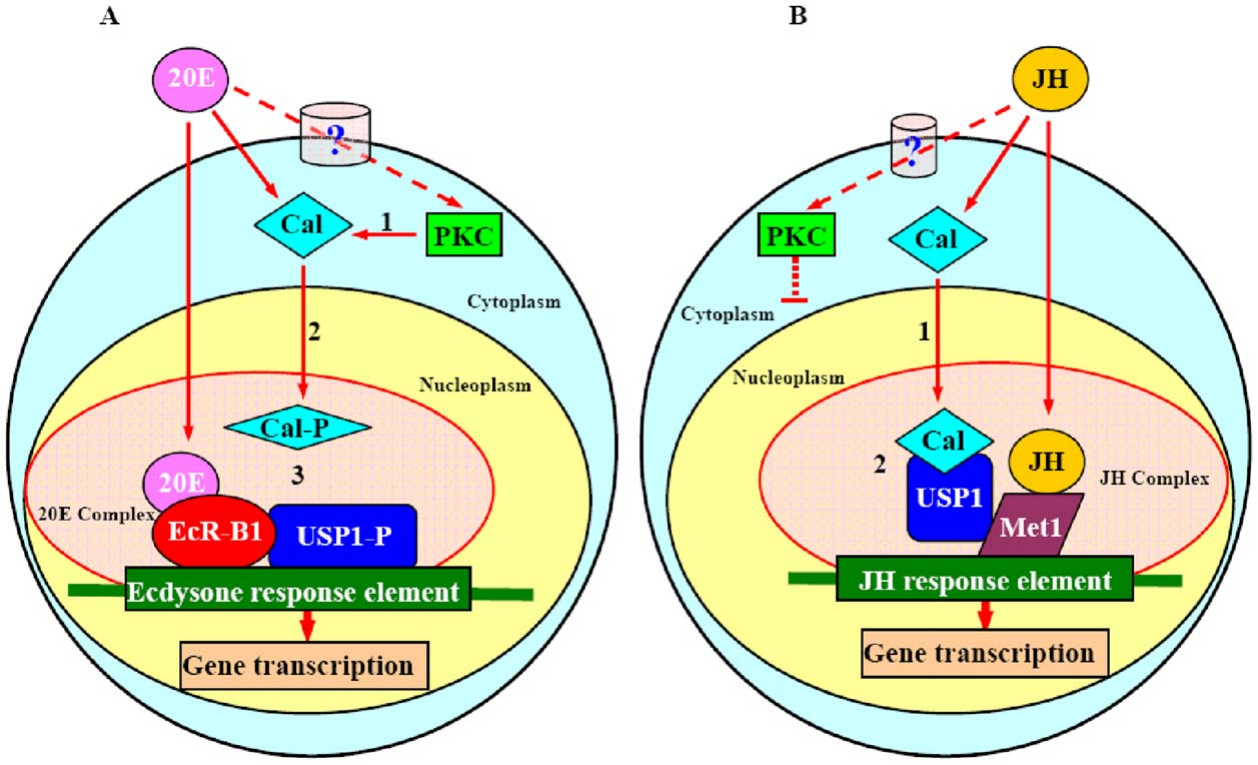
Chart explaining the function of HaCal in the cross talk between 20E and JH pathways. A, 20E pathway: 20E signaling leads HaCal protein phosphorylation by PKC via an unknown membrane pathway (**1**), HaCal is translocated into the nuclei (**2**), phosphorylated HaCal does not bind with phosphorylated USP1 in 20E pathway (**3**). EcR binds 20E and USP [52], [53] and other chaperone protein to form transcription complex, combine the 20E response element initiating the gene transcription [3], [52], [53], [54], [55], [56], [57]. B, JH pathway: Methoprene maintains HaCal non-phosphorylation and translocates HaCal into the nuclei (**1**), non-phosphorylated HaCal binds with non-phosphorylated USP1 (**2**). Met binds JH [20], [58], [59] and interacts with USP and other chaperone proteins [20], [58], and then this complex binds JH response element via Met to initiate JH signaling pathway [59].

In this study, the relatively simpler HaEpi cell line was used to demonstrate the mechanism of 20E and JH interactions. Fig. 9 shows a possible mechanism based on our results, by which *EcR-B1*, *Met1*, *USP1*, and *PKC* are adopted by both 20E and JH pathways in regulating different gene expressions. These factors may play roles by changing their interaction with chaperone proteins, such as *HaCal*, which have different phosphorylation states depending on whether they are induced by 20E or JH.

Upon entering the cytoplasm, 20E triggers PKC signaling through a membrane receptor (still unidentified), and then the background protein of HaCal is quickly transported into the nucleoplasm. The phosphorylated HaCal cannot interact with phosphorylated USP1 [24] but USP can be phosphorylated in the presence of 20E. Thus, USP1 is able to form the 20E receptor complex with EcR-B1 to initiate 20E signaling [39], [40]. In the presence of JH, HaCal is translocated into the nucleoplasm and is maintained in a non-phosphorylated state. The non-phosphorylated HaCal then binds with the non-phosphorylated USP1, which might promote the interaction between USP1 and other proteins such as Met1 in the JH signaling cascade (Fig. 9).

USP1 has been identified as the heterodimeric partner of EcR-B1 and thus critical for the expression of 20E response genes [41]. However, our findings from the RNAi in HaEpi cells showed that, *USP1* was not necessary for 20E or methoprene induction of *HaCal* and *Br-Z2* mRNA. Nevertheless, *USP1* was required for 20E induction of *EcR-B1* and *HR3*. These results may reflect that there are different pathways for 20E inducing gene transcription. *HaCal* was critical for *USP1* transcription in both hormonal inductions, which implicated the importance of *HaCal* in the 20E and JH signaling pathways. Therefore, knockdown of *HaCal* resulted in retarded development, including slowed larval growth and delayed larval molting, and thus *HaCal* is vitally important for insect larval development.

## Materials and Methods

### Animals

*Helicoverpa armigera* larvae were raised on an artificial diet made from powder of wheat germs and soybeans with various vitamins as well as inorganic salts [42] at 28°C with 60%–70% relative humidity and under the light/dark cycles of 14/10 h in an insectarium.

### Semi-quantitative Reverse Transcript PCR (RT-PCR)

Total RNA was isolated from various treated HaEpi cells or insects using Unizol Reagent according to the manufacturer's instructions (Biostar, Shanghai, China). The RNA quality was determined by electrophoresis on agarose gel (1%). Three µg of RNA from each sample was used for the first-strand cDNA synthesis (First Strand cDNA Synthesis Kit, Sangon, China). The resulting cDNAs were used as templates (0.5 ng) in PCR reactions. The cycles of RT-PCR were determined by a gradient increase from 18 to 30 cycles. Three independent experiments were performed with the cDNA templates from various treatments and RNA isolation. The obtained data were statistically analyzed according to the absorbance of the band by Quantity One software (Bio Rad, Hercules, CA, USA). The primers of *HaCal*, *HaEcR-B1*, *HaUSP1*, *HaMet1*, *HaPKC*, *HaBr-Z2, HaGFP*, *HHR3*, *HaJHi*, and *HaRpL27* were adopted in RT-PCR (Table S1).

### Cell Culture

The HaEpi cell line established from the epidermis of *H. armigera* has been well-characterized and proved to present a platform for investigating hormonal regulation in development of lepidopteran insect [11]. The HaEpi cell line was used here in all related experiments. HaEpi cells grew as a loosely attached monolayer and were maintained at 26°C in 25 cm^2^ tissue culture flasks with 4 mL of antibiotic-free Grace′s medium supplemented with 10% heat-inactivated fetal bovine serum. The cells were subcultured weekly to a near confluent monolayer. Cell density was estimated by counting the cells in a suspension aliquot using a hemocytometer under the microscope. All experiments were initiated by seeding flasks with 5×10^5^ cells and cultured under the above-mentioned normal growth conditions for 96 h.

### Hormone treatment on HaEpi cell line

HaEpi cells were maintained to a 90% confluence under the described normal growth conditions above. The cells were cultured for various times after 20E (Sigma, St Louis, MO, USA) or methoprene (Meth, a JH analog) (Sigma, St louis, MO) was added to the cells. The final concentration of both hormones was 1 µM according to previous work [11], [43], [44], [45], [46], [47], [48], [49], [50]. The control cells received an equal volume of dimethylsulfoxide (DMSO), which was used as a solvent for 20E and methoprene.

### Western blot

The cultured cells were collected by scraping and centrifugation, and then lysed with a lysis buffer containing 1% NP-40 to obtain the protein samples. Protein concentration was determined using the Bradford method [51]. Equal amounts of protein (50 µg) were subjected to 15% sodium dodecyl sulfate-polyacrylamide gel electrophoresis (SDS-PAGE) and then electro-transferred onto nitrocellulose membranes. The resulting membranes were incubated for 1 h in a blocking buffer (10 mM Tris-buffered saline solution) containing 2% fat-free milk power at room temperature, and then with the primary anti-HaCal polyclonal antibodies (1:100 dilution in the blocking buffer) at 4°C overnight. Goat anti-rabbit IgG conjugated with horseradish peroxidase (HRP) diluted 1:10,000 in the blocking buffer was adopted as a secondary antibody. 4-Chloro-1-naphthol (4-CN) was used as a HRP substrate for visualizing the peroxidase activity. The quantity of the loaded proteins was controlled by running 2 SDS-PAGE gels simultaneously, one for transferring and the other for Coomassie Brilliant Blue staining.

### Lambda protein phosphatase treatment and protein kinase inhibitor treatment

The procedure for Lambda protein phosphatase (λPP) treatment of the epidermal cell sample was similar to that described previously by Song and Gilbert (1998). In brief, a protein sample from 20E-treated HaEpi cells was incubated for 30 min with 1,000 unit λPP in 50 µL of reaction buffer [500 mM Hepes (N-2-hydroxyethylpiperazine-N-ethane-sulphonicacid), pH 7.5, 1 mM EDTA, and 20 mM MnCl_2_] at 37°C according to the manufacturer's specifications (Millipore, Temecula, CA, USA). At the end of the incubation period, the sample was boiled for 10 min following the addition of SDS sample buffer, and subjected to SDS-PAGE and Western blot analysis. For protein kinase C inhibitor treatment, chelerythrine chloride (CC) (Sigma, St louis, MO, USA) was applied to the cells to obtain a final concentration of 5 µM, then the cells were treated with 20E 30 min later and cultured for an additional 30 min.

### Immunocytochemistry

The hormone treated cells grown on cover slips were fixed with 4% paraformaldehyde in phosphate-buffered saline (PBS) (140 mM NaCl, 2.7 mM KCl, 10 mM Na_2_HPO_4_, 1.8 mM KH_2_PO_4_, pH 7.4) for 15 min. The fixed cells were then incubated in 0.3% Triton-X 100 diluted in PBS for 10 min. After being blocked with 2% fat-free milk powder in PBS for 30 min, the cells were incubated with primary antibodies against the target proteins (1:100 diluted in blocking buffer) for at least 2 h at room temperature. After the washes, the primary antibodies were hybridized with ALEXA 488-labeled goat anti-rabbit secondary antibody (diluted 1:1,000 in blocking buffer) for 1 h. Nuclei were stained with 4′-6′-diamidino-2-phenylindole dihydrochloride (DAPI, 1 µg/mL in PBS) for 10 min. Negative controls were treated similarly, but the pre-immune rabbit serum was used in place of the antisera against the target proteins. The slides were mounted with a mounting medium containing 80% glycerol in PBS. Fluorescence was detected using an Olympus BX51 fluorescence microscope.

### Pull-down assay

A full length cDNA of *HaCal* was cloned in frame into the pGEX-4T-1 vector (Amersham Buckinghamshire, England). This construct was transformed into the competent BL21 (DE3) *Escherichia coli* cells for overexpression with induction by 0.1 mM isopropyl β-D-1-thiogalactopyranoside (IPTG). The cells were then centrifuged at 4,000 g for 10 min, resuspended in 20 mL of PBS containing 0.2% Triton X-100, and sonicated on ice. The GST-HaCal was combined on glutathione Sepharose 4B Resin (Amersham Biosciences AB, Uppsala, Sweden) as bait protein. After being washed with PBS, the GST-HaCal-Sepharose was incubated with soluble recombinant prey protein His-USP1 for 30 min at room temperature. After thorough washing by PBS (274 mM NaCl, 2.7 mM KCl, 20 mM Na_2_HPO_4_, 3.5 mM KH_2_PO_4_, pH 7.0), the protein complex was eluted with an elution buffer (10 mM reduced glutathione, 50 mM Tris-HCl, pH 8.0). The eluted proteins were subjected to SDS-PAGE for analysis. Recombinant soluble cuticle protein (His-CuP) was used as a non-specific binding control.

### Co-immunoprecipitation

Polyclonal antibodies against HaCal were precipitated by ammonium sulfate from the anti-serum with three cycles of alternating saturation, 50% and 30%, and then desalted by dialysis. The purified antibodies were dissolved in 300 µL of coupling buffer containing 0.1 M NaHCO_3_ and 0.5 M NaCl with pH 8.3. CNBr-activated Sepharose 4B (60 mg) (Amersham Biosciences AB, Uppsala, Sweden) was weighed and suspended in 500 µL of 1 mM HCl. After swelling, the powder produced 100 µL of medium. The medium was then washed three times, 5 min each with 1 mM HCl in a 1.5 mL tube. The coupling solution containing antibodies was mixed with the medium in a tube. The mixture was rotated for 1 h at room temperature followed by three washes with at least 5 medium volumes of the coupling buffer. Tris-HCl buffer (0.1 M, pH 8.0) was then used to block the remaining active groups with the medium standing at room temperature for 2 h. The medium was washed with at least three cycles of alternating pH. Each cycle consisted of a wash with 0.1 M acetate buffer, pH 4.0 containing 0.5 M NaCl followed by a wash with 0.1 M Tris-HCl, pH 8.0 containing 0.5 M NaCl.

Proteins were extracted from the hormone-treated cells (by both 20E and methoprene for 6 h respectively) using 0.1 M Tris-HCl buffer, pH 8.0 containing 0.2 M NaCl, 0.5% NP-40, and harvested by centrifugation at 16,000 g for 20 min at 4°C. The supernatant (500 µL) was added to the prepared antibodies-Sepharose mixture with a gentle stir at 4°C overnight. The beads complex were harvested by centrifugation and then triple washed with 0.1 M Tris-HCl buffer, pH 8.0 containing 0.5 M NaCl. The bound proteins were eluted from the Sepharose beads by adding 200 µL of 0.1 µM glycine buffer (pH 2.5). The resulting eluent was finally added with 10 µL of 1 M Tris (pH 8.0) to neutralize the low pH. In the control group, the non-hormone treated cell lysate was incubated with the prepared medium of antibodies-Sepharose, with the bound complexes being eluted as described above. The eluted proteins were detected by Western blot using antibodies against the target proteins.

### RNAi in the HaEpi cell line

The MEGAscript^TM^ RNAi kit (Ambion, Austin, Texas, USA) was used to generate dsRNAs corresponding to *HaCal*, *HaEcR-B1*, *HaUSP1*, *HaMet1*, *HaBr-Z2*, and *HaPKC.* The ssRNA were transcribed at 37°C for over 4 h from PCR templates of the genes (the PCR primers are in Table S1). The dsRNAs were produced by mixing equivalent amounts of complementary ssRNA at 75°C for 5 min followed by slow cooling to room temperature. DNase I and RNase A were used to remove DNA and ssRNA from the dsRNA solutions. The dsRNAs were precipitated with ethanol on acid condition and finally dissolved in nuclease-free water. dsRNA of GFP (green fluorescent protein) was synthesized and used as a non-specific RNA interference control. The concentration of dsRNA was determined by spectrophotometry at 260 nm. After quality determination by 1% agarose electrophoresis, dsRNAs were aliquoted and stored at −20°C ready for experiment.

For dsRNA transfection to the cell line, HaEpi cells were seeded in 6-well plates at 5×10^5^ per well. A lipophilic transfection reagent, Lipofectamine 2000 (Invitrogen, Calsbad, CA, USA) was employed for dsRNA transfection according to the manufacturer′s instructions. The final concentration of dsRNA was 3 µg/mL in the medium. After incubation at 26°C for 10 h post dsRNA transfection, the cells were rinsed and then replenished with a medium containing 1 µM 20E for another 12 h of incubation [49]. The RNA was finally extracted from the cells for RT-PCR analysis.

### RNAi in larvae via bacterial feeding

Full length of HaCal was cloned by PCR using HaCalERNAiF-*Not*I (tactcagcggccgcatgggcgactatcgtgcg) and HaCalERNAiR-*Pst*I (tactcactgcagttacatctgtcgcctggtg) primers. The PCR products were inserted into the RNAi vector pPD129.36. The constructed plasmid was then transformed into competent HT115 (DE5) bacteria. Single colony was picked from the plate and grown with shaking in Luria-Bertani (LB) medium (16 mg/mL tryptone powder, 10 mg/mL yeast extraction, and 5 mg/mL sodium chloride with 100 µg/mL carbenicillin and 12.5 µg/mL tetracycline) at 37°C for 14 h. The bacteria were then subcultured with 100-fold dilution in a LB medium containing carbenicillin and tetracycline at 37°C for 3 h. In order to induce the synthesis of dsRNA in bacteria, the culture was added with IPTG to a final concentration of 0.4 mM and incubated for an additional 4 h at 37°C with shaking. The dsRNA was extracted using Unizol Reagent according to the manufacturer's instructions. The quality of dsRNA was determined by electrophoresis on 1% agarose gel. To prepare the feeding bacteria expressing dsRNA, 200 mL of IPTG-induced culture was used to collect bacteria cells by centrifugation at 4,000 g for 10 min, and then resuspended in 1 mL sterile water for *H. armigera* feeding assay.

The artificial diet was cut into small pieces with relatively similar sizes of about 10 mm×10 mm×2 mm. The bacteria expressing dsRNA for HaCal or expressing control dsRNA for GFP were suspended in sterile PBS and overlaid onto each piece of the prepared diet, respectively. The newly hatched *H. armigetra* larvae were reared on one piece of the diet in a controlled chamber as groups until the 4th instar. The larvae were then reared individually on one piece of the diet once the larvae entered the 5th instar. The diet was refreshed daily. Each treatment contained 30 larvae and made three replicates for statistical analysis. The process of insect development, molting, and metamorphosis were tracked daily. Each individual was weighed using an electronic balance (0.0001 g) (Sartorius, Goettingen, Germany) at given developmental stages. Total RNA of epidermis, midgut, fat body, and haemolymph from the larvae of three typical developmental stages were extracted for RT-PCR analysis. The occurred phenotype was photographed using a Canon (Power shot A 610) digital camera.

## Supporting Information

Figure S1 Hormone regulation on *H. armigera* hormone receptor 3 (*HHR3*) (A) and juvenile hormone inducible gene (*JHi*) (B) in HaEpi cells, checked by RT-PCR. 20E, methoprene was added to cells to a final concentration of 1 µM. The cells were then cultured for 0.5 h, 1 h, 2 h, 4 h and 6 h, respectively. Blue line denotes the trend of 20E regulation; Red line denoted the trend of methoprene regulation; Black line denoted the trend of 20E plus methoprene regulation. The values are mean ± S.D. (*n* = 3).
(TIF)

Figure S2 The statistic analysis of RT-PCR results of RNAi experiments during 20E signaling (A) or JH signaling (B). The values are mean ± S.D. (*n* = 3). *denotes significant difference (*p*<0.05, by student *t* test).
(TIF)

Figure S3 The statistic analysis of RT-PCR results of feeding RNAi experiments in larvae. Epidermis of the larvae ingesting *dsHaCal* at 5F, 5M and 6 W stages was adopted to check the transcript level of *EcR-B1*, *USP1*, *Met1*, *PKC*, *Br-Z2*, *HR3* and *JHi* after *HaCal* was silenced. The values are mean ± S.D. (*n* = 3). *denotes significant difference (*p*<0.05, by student *t* test).
(TIF)

Figure S4 Nucleotide and deduced amino acid sequence of HaCal. Amino acid residues in the shadow indicated calponin homologue domain (Chd) (aa 27-129). The putative protein kinase C phosphorylation sites (aa 39, 73, 80, 128, 171, 177 and 183) were denoted by black solid triangle.
(TIF)

Figure S5 Multiple alignments of HaCal with other calponin homolog domain containing proteins. Calponin of *H. armigera* (HM490090), Calponin of *Aedes aegypti* (Aa-Cal, 001652323), Chd64 of *D. melanogaster* (Dm-Chd64, NP_647860), Chd64 of *Apis mellifera* (Am- Chd64), Transgelin of *B. mori* (Bm-Tran, NP_001040372), Transgelin of *T. castaneum* (Tc-Tran, XP_975100), Transgelin of *Homo sapiens* (Hs-Tran, NP_003177), Calponin of *Homo sapiens* (Hs-Cal, BAA04231). Shadow in black, identity = 100%. Shadow in gray, identity≥80%. Shadow in grayish, identity ≥ 60%. The numbers on the right indicated the amino acid position of different sequences.
(TIF)

Figure S6 Bootstrap consensus phylogenetic tree analysis of HaCal with other calponin homolog domain containing proteins. The sequences (with GENBANK accession number) included: Chd64 of *A. mellifera*, Transgelin of *T. castaneum*, Transgelin of *B. mori*, Calponin of *A. aegypti*, Chd64 of *D. melanogaster*, Calponin of *H. armigera*, Chd64 of *Hydra magnipapillata* (Hm-Chd64, XP_002161847), Calponin of *H. sapiens*, Calponin of *Mus musculus* (Mm-Calponin, AAI38865), Transgelin of *H. sapiens* and Transgelin of *M. musculus* (Mm-Transgelin, NP_035656). The numbers on the branches represented bootstrap values (%) for 1,000 replicates. Branch lengths are proportional to the number of amino acid substitutions. The scale bar on the tree represents the branch length equivalent to 0.1 amino acid changes per residue.
(TIF)

Table S1 Primer list for RNAi and RT-PCR.
(DOC)
