# Supplementary figures and images for: The Participation of Calponin in the Cross Talk between 20-Hydroxyecdysone and Juvenile Hormone Signaling Pathways by Phosphorylation Variation
Source: PLoS One. 2011 May 19;6(5):e19776. doi: 10.1371/journal.pone.0019776 (PMC3098250; doi:10.1371/journal.pone.0019776)

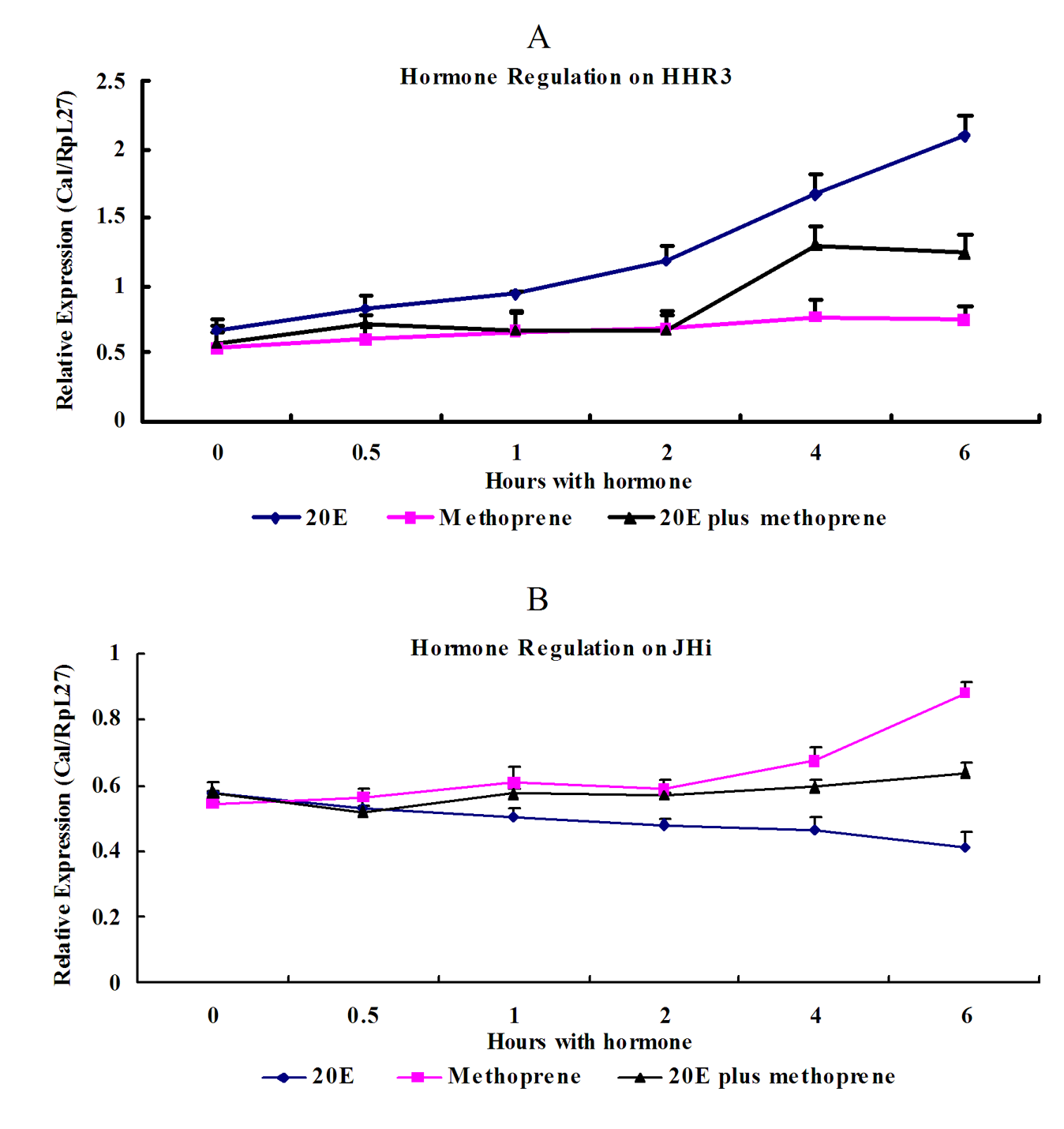

Supplement: Figure S1 — Hormone regulation on H. armigera hormone receptor 3 (HHR3) (A) and juvenile hormone inducible gene (JHi) (B) in HaEpi cells, checked by RT-PCR. 20E, methoprene was added to cells to a final concentration of 1 µM. The cells were then cultured for 0.5 h, 1 h, 2 h, 4 h and 6 h, respectively. Blue line denotes the trend of 20E regulation; Red line denoted the trend of methoprene regulation; Black line denoted the trend of 20E plus methoprene regulation. The values are mean ± S.D. (n = 3). (TIF) [file pone.0019776.s001.tif]

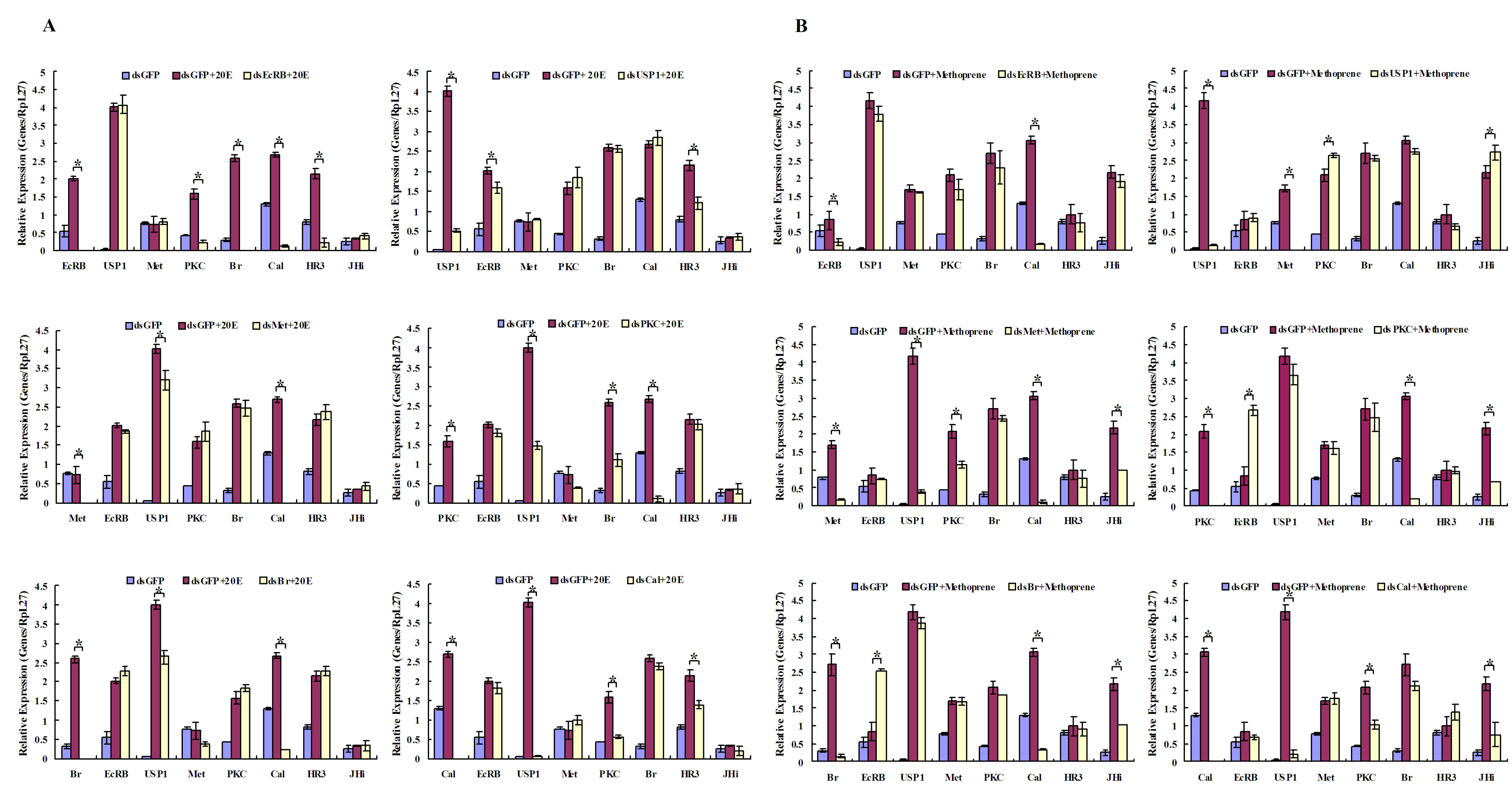

Supplement: Figure S2 — The statistic analysis of RT-PCR results of RNAi experiments during 20E signaling (A) or JH signaling (B). The values are mean ± S.D. (n = 3). *denotes significant difference (p<0.05, by student t test). (TIF) [file pone.0019776.s002.tif]

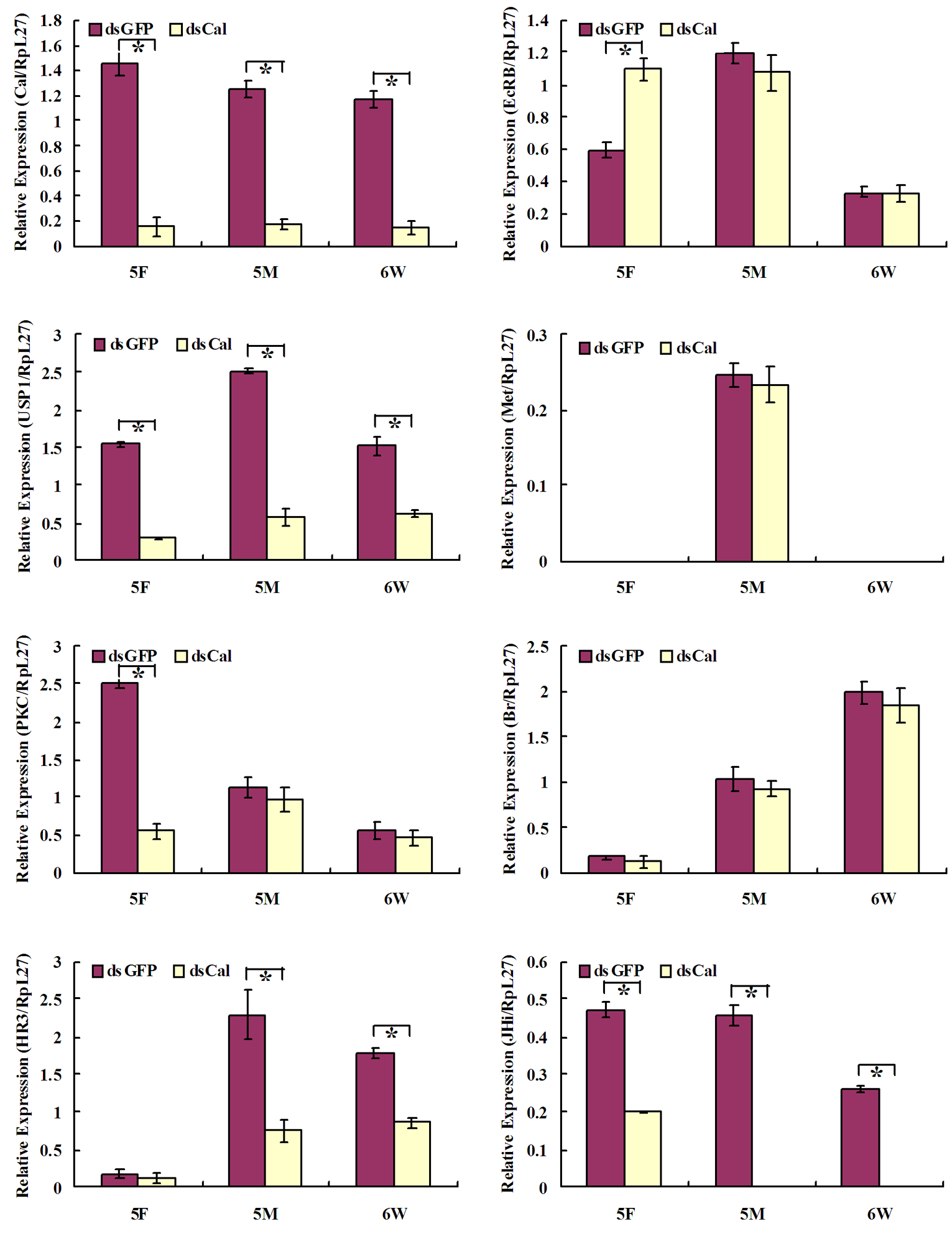

Supplement: Figure S3 — The statistic analysis of RT-PCR results of feeding RNAi experiments in larvae. Epidermis of the larvae ingesting dsHaCal at 5F, 5M and 6 W stages was adopted to check the transcript level of EcR-B1, USP1, Met1, PKC, Br-Z2, HR3 and JHi after HaCal was silenced. The values are mean ± S.D. (n = 3). *denotes significant difference (p<0.05, by student t test). (TIF) [file pone.0019776.s003.tif]

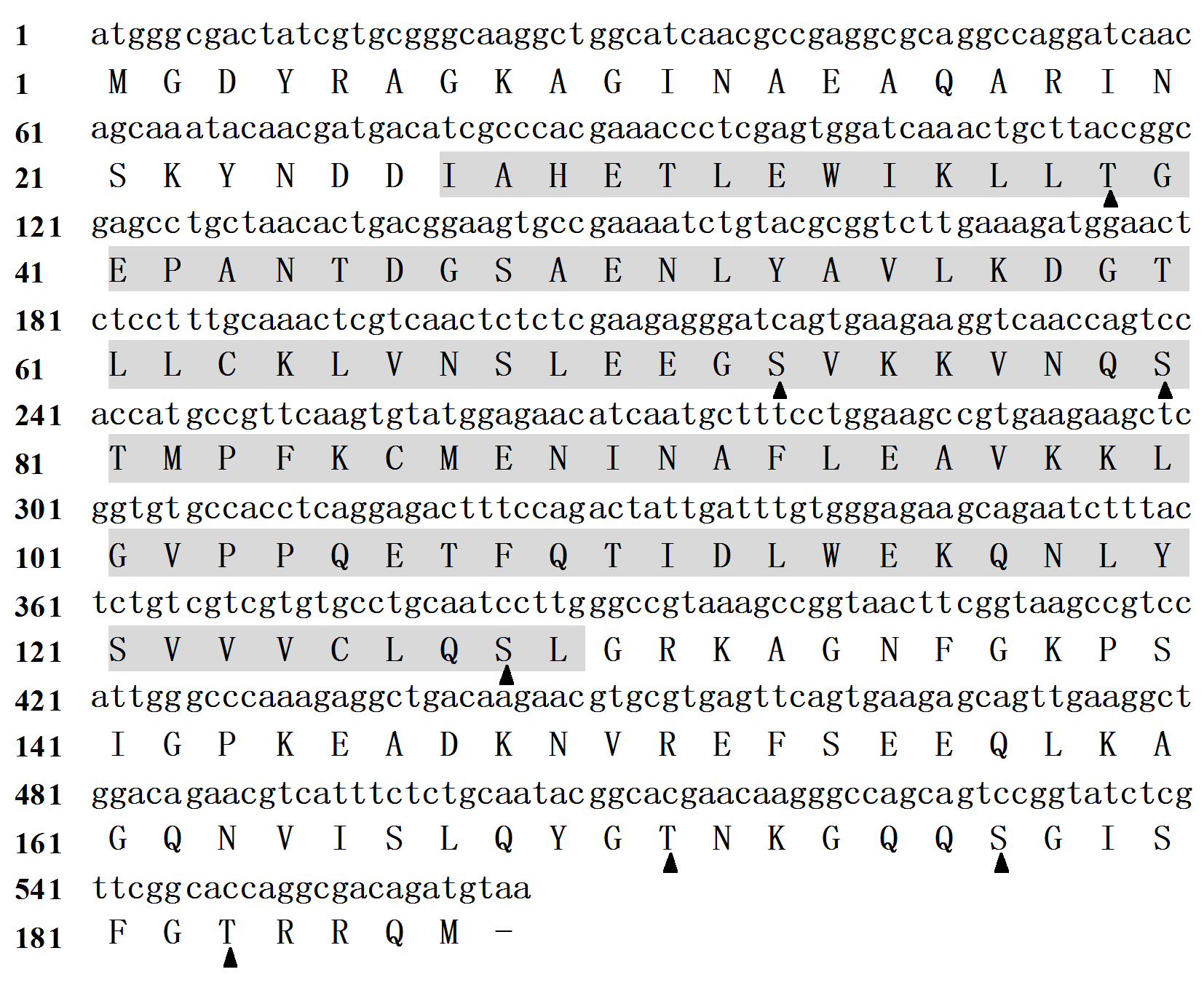

Supplement: Figure S4 — Nucleotide and deduced amino acid sequence of HaCal. Amino acid residues in the shadow indicated calponin homologue domain (Chd) (aa 27-129). The putative protein kinase C phosphorylation sites (aa 39, 73, 80, 128, 171, 177 and 183) were denoted by black solid triangle. (TIF) [file pone.0019776.s004.tif]

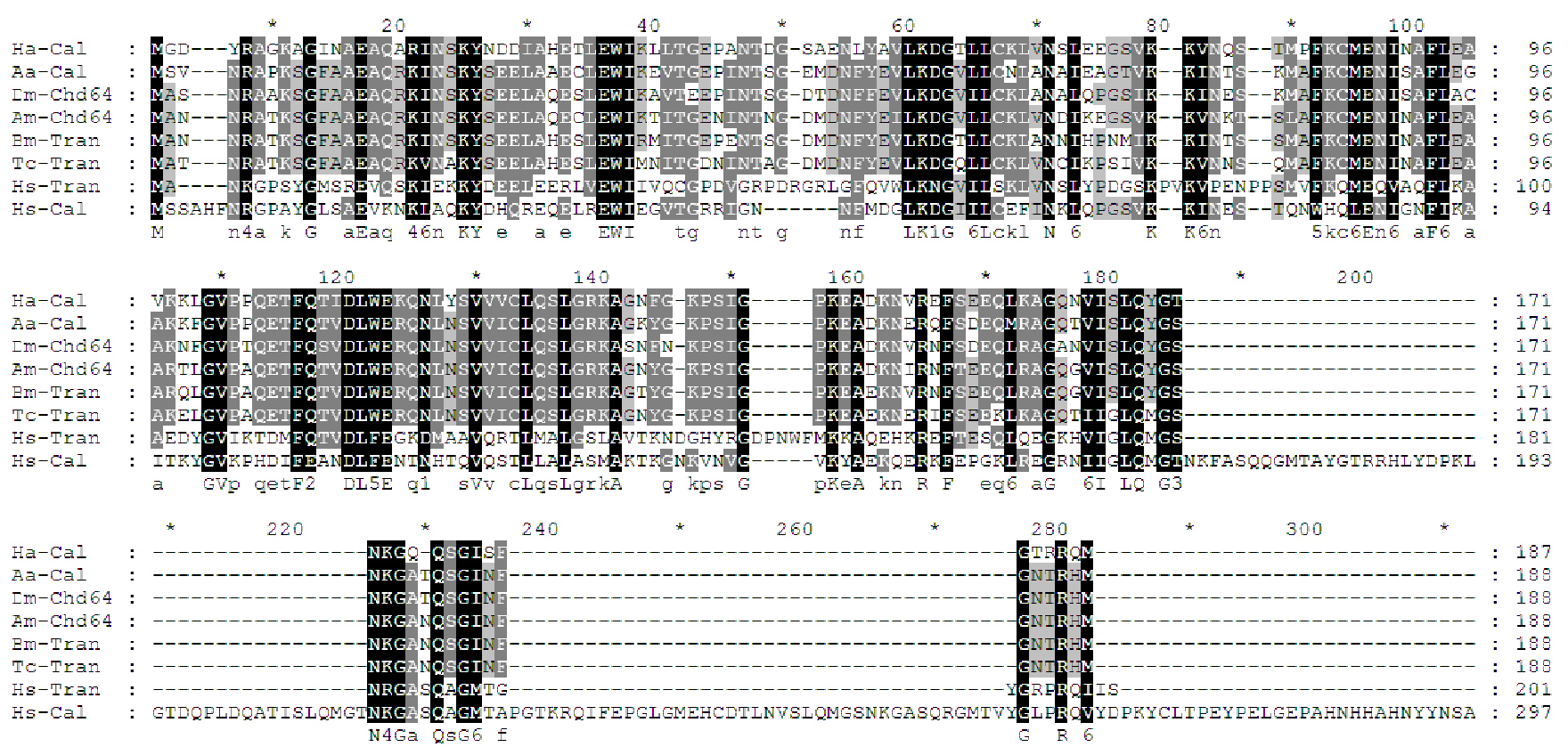

Supplement: Figure S5 — Multiple alignments of HaCal with other calponin homolog domain containing proteins. Calponin of H. armigera (HM490090), Calponin of Aedes aegypti (Aa-Cal, 001652323), Chd64 of D. melanogaster (Dm-Chd64, NP_647860), Chd64 of Apis mellifera (Am- Chd64), Transgelin of B. mori (Bm-Tran, NP_001040372), Transgelin of T. castaneum (Tc-Tran, XP_975100), Transgelin of Homo sapiens (Hs-Tran, NP_003177), Calponin of Homo sapiens (Hs-Cal, BAA04231). Shadow in black, identity = 100%. Shadow in gray, identity≥80%. Shadow in grayish, identity ≥ 60%. The numbers on the right indicated the amino acid position of different sequences. (TIF) [file pone.0019776.s005.tif]

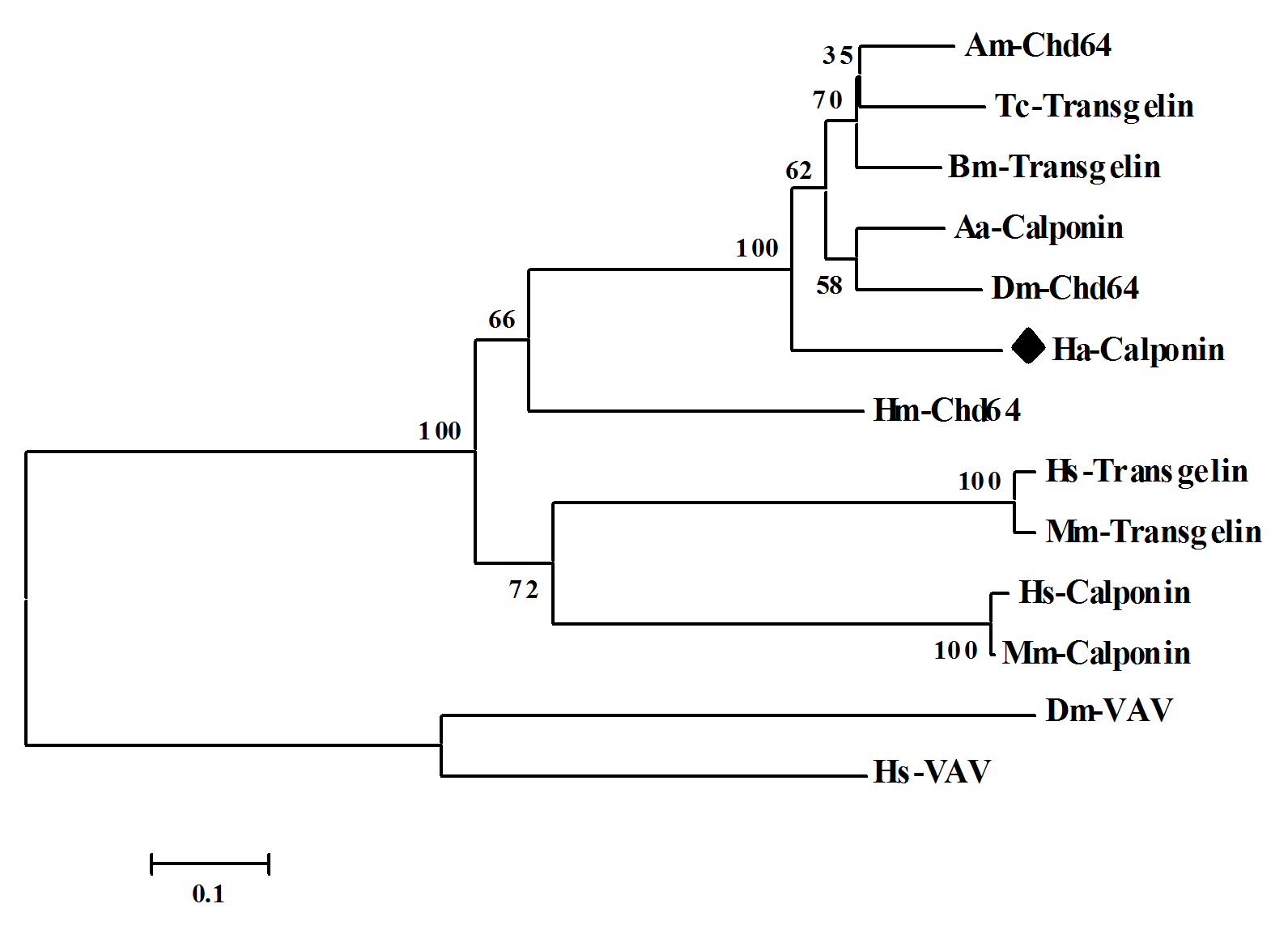

Supplement: Figure S6 — Bootstrap consensus phylogenetic tree analysis of HaCal with other calponin homolog domain containing proteins. The sequences (with GENBANK accession number) included: Chd64 of A. mellifera, Transgelin of T. castaneum, Transgelin of B. mori, Calponin of A. aegypti, Chd64 of D. melanogaster, Calponin of H. armigera, Chd64 of Hydra magnipapillata (Hm-Chd64, XP_002161847), Calponin of H. sapiens, Calponin of Mus musculus (Mm-Calponin, AAI38865), Transgelin of H. sapiens and Transgelin of M. musculus (Mm-Transgelin, NP_035656). The numbers on the branches represented bootstrap values (%) for 1,000 replicates. Branch lengths are proportional to the number of amino acid substitutions. The scale bar on the tree represents the branch length equivalent to 0.1 amino acid changes per residue. (TIF) [file pone.0019776.s006.tif]
