# Supplementary material for: The Participation of Calponin in the Cross Talk between 20-Hydroxyecdysone and Juvenile Hormone Signaling Pathways by Phosphorylation Variation
Source: PLoS One. 2011 May 19;6(5):e19776. doi: 10.1371/journal.pone.0019776 (PMC3098250; doi:10.1371/journal.pone.0019776)
Supplement: Table S1 — Primer list for RNAi and RT-PCR. (DOC) [file pone.0019776.s007.doc]

Table 1. Primer sequences for RNAi and RT-PCR

| Primer name | (5→3) nucleotide sequence |
| --- | --- |
| *EcR-B1* T7F | gcgtaatacgactcactataggcgctggtataacaacggagga |
| *EcR-B1* T7R | gcgtaatacgactcactataggagctggagacaactcctcacg |
| *EcR-B1* F | cgctggtataacaacggagga |
| *EcR-B1* R | agctggagacaactcctcacg |
| *USP1* T7F | gcgtaatacgactcactataggaagggctcctggaacgaa |
| *USP1* T7R | gcgtaatacgactcactataggataggcggtgcgtggttg |
| *USP1* F | aagggctcctggaacgaa |
| *USP1* R | ataggcggtgcgtggttg |
| *Met1* T7F | gcgtaatacgactcactataggtcccgtatcattgcagaa |
| *Met1* T7R | gcgtaatacgactcactatagggtagctcttgagggtaaat |
| *Met1* F | tcccgtatcattgcagaa |
| *Met1* R | gtagctcttgagggtaaat |
| *PKC* T7F | gcgtaatacgactcactataggg |
| *PKC* T7R | gcgtaatacgactcactataggg |
| *PKC* F | actacgccgtgaagtgtttg |
| *PKC* R | agccgctgaatggactctgg |
| *Br-Z2* T7F | gcgtaatacgactcactataggatggctgatcaattctgttta |
| *Br-Z2* T7R | gcgtaatacgactcactatagggttcggtgaagagaaattttc |
| *Br-Z2* F | atggctgatcaattctgttta |
| *Br-Z2* R | gttcggtgaagagaaattttc |
| *HaCal* T7F | gcgtaatacgactcactatagggatgggcgactatcgtgcg |
| *HaCal* T7R | gcgtaatacgactcactatagggttacatctgtcgcctggtg |
| *HaCal* F | atgggcgactatcgtgcg |
| *HaCal* R | ttacatctgtcgcctggtg |
| *GFP* T7F | gcgtaatacgactcactataggtggtcccaattctcgtggaac |
| *GFP* T7R | gcgtaatacgactcactataggcttgaagttgaccttgatgcc |
| *GFP* F | tggtcccaattctcgtggaac |
| *GFP* R | cttgaagttgaccttgatgcc |
| *JHi* F | gaagtcagcggagaacag |
| *JHi* R | cagtcataatacggtgggt |
| *HR3* F | aagggtttcttcaggcgatc |
| *HR3* R | gttggtatttgcgtgtgcttc |
| *RpL27* F | acaggtatccccgcaaagtgc |
| *RpL27* R | gtccttggcgctgaacttctc |
| *HaCal*ERNAiF | tactcagcggccgcatgggcgactatcgtgcg |
| *HaCal*ERNAiR | tactcactgcagttacatctgtcgcctggtg |
